# Supplementary material for: Experimental–numerical method for calculating bending moments in swimming fish shows that fish larvae control undulatory swimming with simple actuation
Source: PLoS Biol. 2020 Jul 22;18(7):e3000462. doi: 10.1371/journal.pbio.3000462 (PMC7481021; doi:10.1371/journal.pbio.3000462)
Supplement: S1 Text — In this supporting information, we provide the detailed mathematical background of the methods used in the article. In addition, we show the results of the validation performed on these methods. S1 Text contains five figures. Fig S1 illustrates the beam representation of the fish body for computation of bending moments. Fig S2 illustrates the reference data used for the internal forces and moments reconstruction. Fig S3 shows the comparison between reference and reconstructed internal forces and moments. Fig S4 shows the convergence of the immersed boundary solver. Finally, Fig S5 shows the comparison between the validated solver and the final choice of the settings of the immersed boundary solver. (PDF) [file pbio.3000462.s001.pdf]

Experimental–numerical method for calculating bending moments in swimming fish shows that fish larvae control undulatory swimming with simple actuation

Supporting Information

Cees J. Voesenek[1], *Gen Li*[2], *Florian T. Muijres*[1], *Johan L. van Leeuwen*[1]

In the main article, we present a method for reconstructing internal bending moments of swimming fish from high-speed video data. First, we reconstruct the 3D motion of the fish from multi-camera high-speed video. The automated method we developed for this has been described in detail previously[1], we will not go into further detail in this Supporting Information. We then use these kinematics to calculate the resultant fluid dynamic forces. Finally, we combine these with the 3D kinematics in a beam model of the fish to calculate the resultant internal forces and moments.

In this Supporting Information, we provide the detailed mathematical background of the methods used in the article. In addition, we show the results of the validation performed on these methods.

1 Calculating fluid forces from kinematics

We refer to the section "Calculating fluid-force distribution" in the Materials and Methods section of main text for details on the computational method we used for solving the Navier-Stokes equations around reconstructed 3D swimming kinematics.

Flow fields calculated numerically were post-processed to extract the fluid force distribution on the skin of the fish with a custom Python 3 program. In this program, we interpolated[2] the pressure and velocity gradients to a triangulated surface slightly offset from the fish skin. These were then used to calculate the local stress on each triangular face as:

$$\sigma_{\text{pressure}} = -p\mathbf{n}; \tag{S1}$$

$$\sigma_{\text{friction}} = \boldsymbol{\tau}\mathbf{n}, \tag{S2}$$

where p is the pressure, $\boldsymbol{\tau}$ is the shear stress tensor, and $\mathbf{n}$ is the outward facing normal of the face. Under the assumption of a Newtonian fluid, the shear stress tensor is defined as:

$$\boldsymbol{\tau} = \mu \begin{bmatrix} 2\frac{\partial u}{\partial x} & \frac{\partial u}{\partial y} + \frac{\partial v}{\partial x} & \frac{\partial u}{\partial z} + \frac{\partial w}{\partial x} \\ \frac{\partial u}{\partial y} + \frac{\partial v}{\partial x} & 2\frac{\partial v}{\partial y} & \frac{\partial v}{\partial z} + \frac{\partial w}{\partial y} \\ \frac{\partial u}{\partial z} + \frac{\partial w}{\partial x} & \frac{\partial v}{\partial z} + \frac{\partial w}{\partial y} & 2\frac{\partial w}{\partial z} \end{bmatrix}, \tag{S3}$$

where μ is the dynamic viscosity, and u, v, w are the velocity components in respectively x-, y-, and z-direction. These surface stress distributions were then grouped into segments along the fish to calculate the local net fluid force in the moving reference frame.

[1]Experimental Zoology Group, Wageningen University, PO Box 338, NL-6700 AH Wageningen, The Netherlands.

[2]Department of Mathematical Science and Advanced Technology, Japan Agency for Marine-Earth Science and Technology (JAMSTEC), 3173-25, Showa-machi, Kanazawa-ku, Yokohama, Japan.

2 Equations of motion

We use the reconstructed kinematics and fluid-dynamic forces to the internal forces and moments by modelling the fish as a bending 'beam'. In this section, we show the derivation of the equations of motion for this large-deformation beam representation of the fish.

2.1 Deriving the equations of motion

We model the fish as a beam with varying cross-sections, undergoing arbitrarily large deformation. Plane cross-sections are assumed to remain plane and perpendicular to the neutral line (no shear deformation), but axial deformation is allowed. Although the motion we tracked from the video is three-dimensional, we assume that the fish deforms in a single plane. Therefore, we can use a two-dimensional beam model to represent the deformation of the fish, under a suitable coordinate transformation. In summary, we model the fish as a beam undergoing large bending deformations in two dimensions.

We describe the deformation of the beam with the displacement of each infinitesimal beam element with respect to the reference configuration (Fig. S1A). It is defined as a function $\boldsymbol{\xi}(s,t) = \big(\xi(s,t), \eta(s,t)\big)$ of a parameter s along the length of the beam, and the time t. The position of the central axis at each point s is given by:

$$\big(x(s,t), y(s,t)\big) = \big(x_0(s) + \xi(s,t), y_0(s) + \eta(s,t)\big), \tag{S4}$$

where $(x_0(s), y_0(s))$ is the reference configuration of the beam. We define the reference configuration as a straight beam aligned with the positive x-axis, so the position becomes:

$$\big(x(s,t), y(s,t)\big) = \big(s + \xi(s,t), \eta(s,t)\big), \tag{S5}$$

This displacement results in a local deformation angle $\theta(s,t)$ for each beam element (Fig. S1A). It can be calculated from the displacements with:

$$\theta = \arctan\left(\frac{\frac{\partial \eta}{\partial s}}{1 + \frac{\partial \xi}{\partial s}}\right). \tag{S6}$$

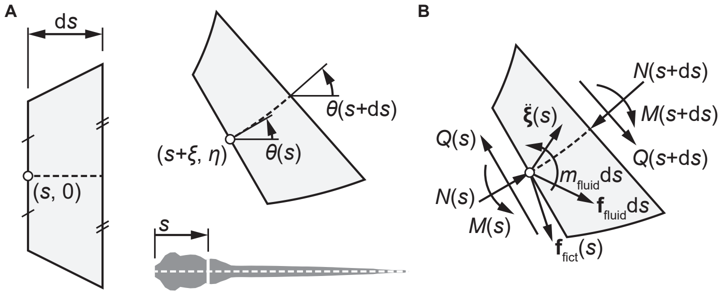

Fig. S1. Beam representation of the fish body for computation of bending moments. (A) Geometric definitions of a beam element $\mathrm{d}s$ at a position s along the body, in reference (left) and deformed (right) configuration. We consider an infinitesimal element at $(s, 0)$ in the reference configuration, that is displaced by (ξ, η), and rotated by an angle θ. (B) Free body diagram of a beam element $\mathrm{d}s$, with axial force N, shear force Q, bending moment M, net fictitious force distribution $\mathbf{f}_{\mathrm{fict}}$, net fluid force distribution $\mathbf{f}_{\mathrm{fluid}}$, net in-plane fluid moment distribution m_{fluid}, and acceleration $\ddot{\boldsymbol{\xi}}$.

To derive the equations of motion, we consider a free body diagram of an infinitesimal beam element of length $\mathrm{d}s$ (Fig. S1B). The force balance in x- and y-direction for a beam ele-

ment $\mathrm{d}s$ can be expressed as:

$$\begin{aligned}\rho_{\text{fish}} A(s)\,\mathrm{d}s\,\tfrac{\partial^2 \xi}{\partial t^2}(s,t) = {} & N(s,t)\cos\big(\theta(s,t)\big) - N(s+\mathrm{d}s,t)\cos\big(\theta(s+\mathrm{d}s,t)\big)\\ & - Q(s,t)\sin\big(\theta(s,t)\big) + Q(s+\mathrm{d}s,t)\sin\big(\theta(s+\mathrm{d}s,t)\big)\\ & + f_{x,\text{muscle}}(s,t)\,\mathrm{d}s + f_{x,\text{fluid}}(s,t)\,\mathrm{d}s + f_{x,\text{fict}}(s,t)\,\mathrm{d}s;\end{aligned} \tag{S7}$$

$$\begin{aligned}\rho_{\text{fish}} A(s)\,\mathrm{d}s\,\tfrac{\partial^2 \eta}{\partial t^2}(s,t) = {} & N(s,t)\sin\big(\theta(s,t)\big) - N(s+\mathrm{d}s,t)\sin\big(\theta(s+\mathrm{d}s,t)\big)\\ & + Q(s)\cos\big(\theta(s,t)\big) - Q(s+\mathrm{d}s,t)\cos\big(\theta(s+\mathrm{d}s,t)\big)\\ & + f_{y,\text{muscle}}(s,t)\,\mathrm{d}s + f_{y,\text{fluid}}(s,t)\,\mathrm{d}s + f_{y,\text{fict}}(s,t)\,\mathrm{d}s,\end{aligned} \tag{S8}$$

where ρ_{fish} is the beam density, A is the beam cross-sectional area, N is the internal normal force, Q is the internal shear force, $f_{x,\text{muscle}}$, $f_{y,\text{muscle}}$ are the muscle forces in x- and y-direction, $f_{x,\text{fluid}}$, $f_{y,\text{fluid}}$ are the external fluid forces in x- and y-direction, and $f_{x,\text{fict}}$, $f_{y,\text{fict}}$ are the fictitious forces in x- and y-direction (from the non-inertial reference frame, see section 2.2).

The moment balance (counter-clockwise positive) about the point on the neutral line at $s + \mathrm{d}s$ is given by:

$$\begin{aligned}\rho_{\text{fish}} I(s)\,\mathrm{d}s\,\tfrac{\partial^2 \theta}{\partial t^2}(s,t) = {} & M(s,t) - M(s+\mathrm{d}s,t) - Q(s,t)\mathrm{d}s\\ & + m_{\text{muscle}}(s,t)\,\mathrm{d}s + m_{\text{fluid}}(s,t)\,\mathrm{d}s + m_{\text{fict}}(s,t)\,\mathrm{d}s,\end{aligned} \tag{S9}$$

where I is the distribution of the second moment of area, M is the internal moment, m_{muscle} is the muscle moment per unit length, m_{fluid} is the external fluid moment per unit length, and m_{fict} is the fictitious moment per unit length (see section 2.2).

Dividing equations S7, S8, and S9 by the infinitesimal length $\mathrm{d}s$, applying the definition of a derivative $\frac{\partial \varphi}{\partial s}(s,t) = \frac{\varphi(s+\mathrm{d}s,t)-\varphi(s,t)}{\mathrm{d}s}$, and dropping the explicit $f(s,t)$ notation, yields the equations of motion:

$$\rho_{\text{fish}} A\,\frac{\partial^2 \xi}{\partial t^2} = -\frac{\partial}{\partial s}\big(N\cos\theta\big) + \frac{\partial}{\partial s}\big(Q\sin\theta\big) + f_{x,\text{muscle}} + f_{x,\text{fluid}} + f_{x,\text{fict}}; \tag{S10}$$

$$\rho_{\text{fish}} A\,\frac{\partial^2 \eta}{\partial t^2} = -\frac{\partial}{\partial s}\big(N\sin\theta\big) - \frac{\partial}{\partial s}\big(Q\cos\theta\big) + f_{y,\text{muscle}} + f_{y,\text{fluid}} + f_{y,\text{fict}}; \tag{S11}$$

$$\rho_{\text{fish}} I\,\frac{\partial^2 \theta}{\partial t^2} = -\frac{\partial M}{\partial s} - Q + m_{\text{muscle}} + m_{\text{fluid}} + m_{\text{fict}}. \tag{S12}$$

2.2 Fictitious forces

We reconstructed the motion of the fish from video in three-dimensional space, but described the equations of motion in a two-dimensional plane. However, in the video-tracking method, we assumed that the fish deforms in a single plane. Hence, we can create a coordinate system aligned to this plane and obtain the equations in two dimensions only. We define this head reference frame as fixed to the snout of the fish and rotating along with the stiff head region in the deformation plane. Any point $\hat{\mathbf{x}}$ in world coordinates (denoted with a circumflex) can be expressed in the fish coordinate system at time t as:

$$\mathbf{x} = \mathrm{R}^{\mathrm{T}}(t)\,(\hat{\mathbf{x}} - \hat{\mathbf{x}}_{\text{snout}}), \tag{S13}$$

where $\mathrm{R}(t)$ is the time-dependent rotation matrix expressing the orientation of the snout and $\hat{\mathbf{x}}_{\text{snout}}$ is the position of the snout.

When we transform the motion to the non-inertial fish reference frame, additional equation terms accounting for the effect of the translation and rotation of the frame must be considered.

These additional acceleration terms for any point $\mathbf{x}$ in the rotating reference frame are given by[3]:

$$\mathbf{a}_{\text{add}} = -\hat{\mathbf{a}}_{\text{head}} - 2\hat{\boldsymbol{\omega}} \times \mathbf{v} - \hat{\boldsymbol{\omega}} \times (\hat{\boldsymbol{\omega}} \times \mathbf{x}) - \hat{\boldsymbol{\alpha}} \times \mathbf{x}, \tag{S14}$$

where $\hat{\mathbf{a}}_{\text{head}}$ is the acceleration of the origin, $\hat{\boldsymbol{\omega}}$ is the angular velocity of the rotating coordinate system, $\mathbf{v}$ is the velocity of the considered point, and $\hat{\boldsymbol{\alpha}}$ is the angular acceleration of the rotating coordinate system. Note that the quantities $\hat{\mathbf{a}}_{\text{head}}$, $\hat{\boldsymbol{\omega}}$, and $\hat{\boldsymbol{\alpha}}$ are calculated with respect to the world reference frame, but expressed in the basis vectors of the moving reference frame. The position $\mathbf{x}$ and velocity $\mathbf{v}$ are expressed with respect to the moving reference frame.

These accelerations can be considered as an additional 'fictitious' external force distribution in the moving reference frame. This force distributions is given by

$$\mathbf{f}_{\text{fict}} = \rho A\, \mathbf{a}_{\text{add}}. \tag{S15}$$

These fictitious forces are added to the fluid-dynamic forces to calculate the total external force distribution acting on each beam element.

3 Reconstructing internal forces and moments with inverse dynamics

We reconstructed bending moments from the motion of the fish and its simulated external fluid force distribution, an approach commonly called inverse dynamics. This section describes the optimisation procedure we used to reconstruct the internal forces and moments.

In our inverse dynamics approach, we cannot separate the effects of the active and passive tissues inside the fish: the internal forces and moments that we compute include the effects of both. Considering this, the moment equation becomes:

$$\rho_{\text{fish}} I \, \frac{\partial^2 \theta}{\partial t^2} = -\frac{\partial M^*}{\partial s} - Q + m_{\text{fluid}} + m_{\text{fict}}, \tag{S16}$$

where $\frac{\partial M^*}{\partial s} = \frac{\partial M}{\partial s} - m_{\text{muscle}}$. This combined moment is what we reconstruct from the motion with our optimisation procedure. From here onwards, we drop the asterisk notation and refer to the combined active and passive internal bending moment as the 'bending moment'.

3.1 Optimising internal forces and moments

To calculate the normal force, shear force, and bending moment, we used an optimisation algorithm that determines the best-fitting distributions in space and time. At every point in time, we described the internal forces and moments with a quintic spline[2] along the length of the fish. Values were prescribed at 12 uniformly spaced control points, between which the values were interpolated with the spline. The first and last control point, at respectively $s = 0$ and $s = \ell$, were fixed at a value of 0, to satisfy the boundary conditions of $N(0) = N(\ell) = 0$, $Q(0) = Q(\ell) = 0$, and $M(0) = M(\ell) = 0$ that should hold for the free ends of a beam.

At each time step, we optimised the moment-, shear-, and normal distributions to minimise the deviation from equations S10, S11, S12 at every point along the fish. This deviation was quantified by the residual value that is needed to balance the left- and right-hande side of the equations. We optimised the distributions with the Levenberg-Marquardt algorithm[2], that minimises the squares of the residuals. At every time step, this resulted in a series of control point values describing the internal forces and moments that best satisfy the equations.

3.2 Calculating resultant power

We calculated the resultant power on the fish from two sources: the power exerted on the fluid, and the changes in kinetic energy. Both quantities are computed in the inertial reference frame. We calculated the power per unit length that the fish exerts on the water as:

$$\hat{p}_{\text{fluid}} = -\hat{\mathbf{f}}_{\text{fluid}} \cdot \hat{\mathbf{v}}, \tag{S17}$$

where $\hat{\mathbf{v}}$ is the velocity of the centreline in world coordinates. We negated the power because we are considering the power that the fish exerts on the water, rather than the inverse.

The kinetic energy per unit length at any point in time was calculated as:

$$\hat{e}_{\text{kinetic}} = \frac{1}{2}\rho A||\hat{\mathbf{v}}||^2. \tag{S18}$$

The kinetic power per unit length is the time derivative of the kinetic energy:

$$\hat{p}_{\text{kinetic}} = \frac{\partial \hat{e}_{\text{kinetic}}}{\partial t}. \tag{S19}$$

Note that we do not calculate powers based on the internal forces and moments. We do not separate passive and active effects, which might compensate each other. Hence, the power calculated from the net internal forces and moments does not correspond to the actual power consumption by the active components only. This problem is similar to modelling the power consumption of an aeroplane flying at constant speed without separating thrust and drag sources: because there are no net forces, the net power calculated is zero. However, when the 'active' component (i.e. thrust from the engines) is separated from the 'passive' component (i.e. drag of the aircraft), the computed power consumption is clearly non-zero.

4 Integrating the equations of motion to generate reference data

We integrated the equations of motion to verify whether the derived equations are physically valid and to generate reference data to test our algorithm for reconstructing bending moments. This section explains how we numerically solved the derived non-linear equations of motion.

4.1 Constitutive and kinematic relations

The normal forces and moments can be calculated from the displacements using constitutive equations. To generate the reference data, we assumed a Hookean material, resulting in the following equations:

$$N = -YA\varepsilon; \tag{S20}$$

$$M = -YI\frac{\partial \theta}{\partial s}, \tag{S21}$$

where Y is the Young's modulus. The strain ε can be computed from the displacements as follows:

$$\varepsilon = \sqrt{\left(1 + \frac{\partial \xi}{\partial s}\right)^2 + \left(\frac{\partial \eta}{\partial s}\right)^2} - 1. \tag{S22}$$

These relations complete the set of equations required to calculate the accelerations of each point on the beam with equations S10 and S11.

4.2 Temporal integration

We used the backward Euler method to integrate the beam accelerations to velocities, and velocities to displacements. To calculate the velocities and displacements in x-direction at the time step i, we used:

$$\left(\frac{\partial \xi}{\partial t}\right)_i = \left(\frac{\partial \xi}{\partial t}\right)_{i-1} + \Delta t \left(\frac{\partial^2 \xi}{\partial t^2}\right)_i ; \tag{S23}$$

$$\xi_i = \xi_{i-1} + \Delta t \left(\frac{\partial \xi}{\partial t}\right)_i , \tag{S24}$$

and analogous expressions for η.

4.3 Equation scaling

The governing equations resulted in an ill-conditioned system, caused by large scale differences in the matrix coefficients. This makes a system difficult to solve numerically. To improve this, we scaled the variables such that all coefficients were close to 1. We used the following scaling coefficients, where an asterisk denotes a scaled quantity:

$$\begin{gathered} s^* = \frac{1}{\Delta s} s; \quad t^* = \frac{1}{\Delta t} t; \\ \xi^* = \frac{1}{\Delta s} \xi; \quad \eta^* = \frac{1}{\Delta s} \eta; \quad \theta^* = \theta; \\ N^* = \frac{\Delta t^2}{\rho A \Delta s^2} N; \quad Q^* = \frac{\Delta t^2}{\rho A \Delta s^2} Q; \quad M^* = \frac{\Delta t^2}{\rho I \Delta s} M. \end{gathered} \tag{S25}$$

The scaled equations for temporal integration of ξ then become:

$$\xi^*_i - \xi^*_{i-1} - \left(\frac{\partial \xi^*}{\partial t^*}\right)_i = 0, \tag{S26}$$

and analogously for the other three equations related to integration of ξ and η.

Scaling the force balance in x-direction yields:

$$\frac{\partial^2 \xi^*}{\partial t^{*2}} + \frac{\partial N^*}{\partial s^*} \cos\theta^* - N^* \frac{\partial \theta^*}{\partial s^*} \sin\theta - \frac{\partial Q^*}{\partial s^*} \sin\theta^* - Q^* \frac{\partial \theta^*}{\partial s^*} \cos\theta - \frac{\Delta t^2}{\rho A \Delta s} f_x = 0. \tag{S27}$$

Equivalently, for the force balance in y-direction:

$$\frac{\partial^2 \eta^*}{\partial t^{*2}} + \frac{\partial N^*}{\partial s^*} \sin\theta^* + N^* \frac{\partial \theta^*}{\partial s^*} \cos\theta^* + \frac{\partial Q^*}{\partial s^*} \cos\theta^* - Q^* \frac{\partial \theta^*}{\partial s^*} \sin\theta^* - \frac{\Delta t^2}{\rho A \Delta s} f_y = 0. \tag{S28}$$

The scaled moment balance becomes:

$$\frac{\partial^2 \theta^*}{\partial t^{*2}} + \frac{\partial M^*}{\partial s^*} + \frac{A \Delta s^2}{I} Q^* - \frac{\Delta t^2}{\rho I} m = 0 \tag{S29}$$

Finally, the constitutive and kinematic relations become:

$$N^* + \frac{Y \Delta t^2}{\rho \Delta s^2} \left[\sqrt{\left(1 + \frac{\mathrm{d}\xi^*}{\mathrm{d}s^*}\right)^2 + \left(\frac{\mathrm{d}\eta^*}{\mathrm{d}s^*}\right)^2} - 1 \right] = 0 \tag{S30}$$

$$M^* + \frac{Y \Delta t^2}{\rho \Delta s} \frac{\partial \theta^*}{\partial s^*} = 0 \tag{S31}$$

$$\theta^* - \arctan\left(\frac{\frac{\partial \eta^*}{\partial s^*}}{1 + \frac{\partial \xi^*}{\partial s^*}}\right) = 0 \tag{S32}$$

4.4 Solution method

We integrated the scaled equations of motion with multivariate Newton-Raphson, an iterative solution method for non-linear partial differential equations. Based on the solution at the previous time step, we calculated the vector of residuals for each of the 4 temporal integration relations, 3 force and moment balances, and 3 constitutive and kinematics relations per point along the beam. This led to a vector of $10n_{\mathrm{lon}}$ residuals, with n_{lon} the number of longitudinal points in the beam. To calculate the value for the next iteration, we solved:

$$\mathcal{J}_F(\boldsymbol{\varphi}_i)\big(\boldsymbol{\varphi}_{i+1} - \boldsymbol{\varphi}_i\big) = -F(\boldsymbol{\varphi}_i), \tag{S33}$$

where the subscripts i and $i+1$ denote the current and next iteration, $\mathcal{J}_F$ is the Jacobian of the residuals, F is the vector of residuals, and $\boldsymbol{\varphi}$ is the vector of variables.

We calculated the Jacobian numerically by perturbing each variable with a fixed-step size and then calculating one-sided finite-differences. We solved the system with a direct solver[4].

5 Validating the inverse dynamics methods

This section describes the validation of the internal forces and moments reconstruction, based on reference data and based on a reference CFD simulation.

We assessed the validity of the equations of motion by comparing a simulated beam to experimental results[5]. We then tested the reconstruction of internal forces and moments based on reference data produced by integrating the equations of motion (see section 4).

5.1 Equations of motion

To assess the validity of the derived beam equations, we compared experimental results from Beléndez et al.[5] with our simulation of a cantilever beam. In their study, a 300 mm steel ruler ($Y = 200$ GPa) with rectangular cross-section (width $\times$ height = 30.4 mm $\times$ 0.78 mm) was clamped at one end. The beam was loaded with a point force of 3.92 N in negative y-direction at the unclamped tip, in addition to the distributed gravity load of 1.85 N m^{-1} (total 0.554 N over 300 mm).

We simulated a beam with the same geometry and loading, but in a time-dependent manner. We started with the beam in undeformed configuration, then smoothly ramped the loading from 0 to the reference amplitudes over a period of 5 seconds, and continued simulating for 5 more seconds. The resulting deformation was compared to the experimental reference in Fig. S2A—it overlaps strikingly, providing confidence in the physical validity of the derived equations of motion.

5.2 Calculating internal forces and moments

To check the correctness of the algorithm for reconstructing forces and moments, we generated a simple model of a 'swimmer' by prescribing analytical external forces and moments to a simulated beam of a Hookean material (see section 4). We prescribed the fluid forces as:

$$f_{x,\mathrm{fluid}}(s,t) = A_x \sin(2\pi f t); \tag{S34}$$

$$f_{y,\mathrm{fluid}}(s,t) = A_y \cos(2\pi f t), \tag{S35}$$

and the muscle moment by:

$$m_{\mathrm{muscle}} = A_m \sin(2\pi f t)\left[1 + \sin\left(2\pi \frac{s}{\ell} - \frac{\pi}{2}\right)\right]. \tag{S36}$$

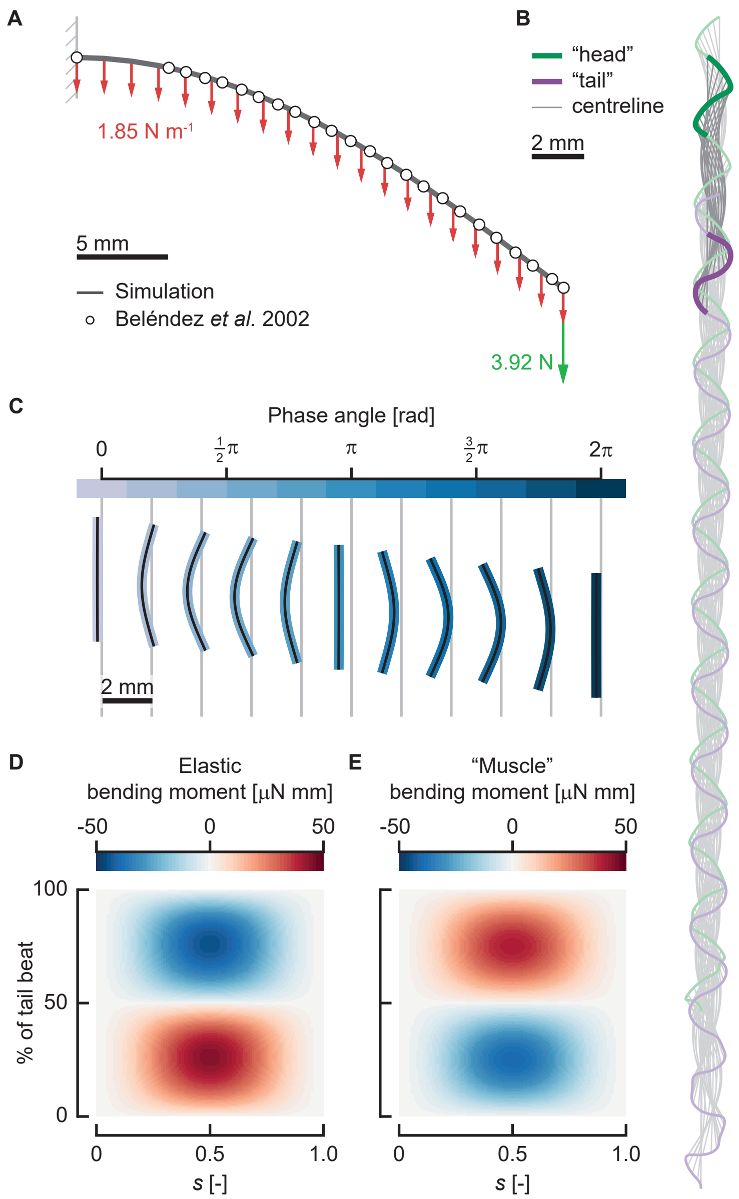

Fig. S2. Reference data for the internal forces and moments reconstruction. (A) Comparison of our simulated beam (solid grey line) with experimental results[5] dots, under a distributed and point load (resp. red and green arrows). (B) Motion of the simulated reference 'swimmer', with the beam centrelines (grey), path of the 'head' (green) and of the 'tail' (purple). The dark grey, green, and purple indicate the cycle selected for further analysis. (C) The motion of the reference 'swimmer' over the selected cycle, centreline (black), beam coloured by phase angle. (D)–(E) The elastic contribution (D) and the 'muscle' contribution (E) to the internal bending moment for the reference 'swimmer'.

The resulting motion is shown in Fig. S2B, demonstrating a swimming-like motion. We selected a single cycle from the motion (Fig. S2C), after the motion had become reliably periodic after 11 cycles. The total internal bending moment, which is what we reconstruct, consisted of an elastic contribution (Fig. S2D), and a contribution from the 'muscle' moment (Fig. S2E).

We reconstructed the total internal forces and moments of the reference 'swimmer' with the method described in section 3, see Fig. S3. The results match well, both qualitatively and quantitatively. Qualitatively, the patterns are similar, showing the same dynamics between the reference and the reconstruction. The shear force and normal force show relatively the largest errors (respectively maximum 9.7% and 7.0% of the peak value), while the error in bending moment is low (maximum 0.9% of the peak value). This shows that our main quantity of interest, the bending moment, can be reliably reconstructed with the proposed method.

6 Validating the method against a reference fluid-dynamics solver

As a reference to compare the computational fluid dynamics (CFD) results of IBAMR, we used an extensively validated numerical method for fish free swimming[6,7,8]. The curvature of the fish was prescribed similar to Li et al.[7], by a travelling curvature wave (rather than an amplitude wave in the original reference) with an experimental curvature amplitude envelope of a 3 days post fertilisation zebrafish larva. The frequency was 50 Hz, the fish length 3.8 mm, the water density 1000 kg m^{-3}, and the dynamic viscosity 0.8301 mPa s. The motion of the fish was calculated based on the fluid dynamic forces, resulting in a free-swimming fish. We used the resulting fluid force distributions and motion to calculate internal forces and moments.

We prescribed the same motion with a custom-developed add-on to the open-source immersed boundary method IBAMR[9], see section 1. Note that we did not integrate the motion of the fish, but prescribed the position of the fish surface directly at all time points. For the results from IBAMR, we also calculated the internal forces and moments to compare to the reference from the validated solver.

6.1 Influence of the surface offset

We calculated the force distributions on the fish by interpolating quantities from the CFD flow field to a triangulated surface of slightly offset from the skin of the fish. The amplitude of these forces is dependent on this offset, related to the accuracy of the interpolation of the pressure and velocity gradients. Due to the immersed boundary approach, there is also a flow field inside the fish, but this is not physically relevant for the force calculations. If this is taken into account in the interpolation, errors in the force distribution will occur.

Fig. S4A shows the effect of the surface offset on the accuracy of the bending moment reconstruction. The optimal distance for the offset surface for a simulation with a finest mesh size of 15 μm (the final selected mesh size) was found to be 20 μm. This distance guaranteed that the flow field was interpolated from only cells outside the body, but was close enough to accurately reconstruct the frictional forces.

6.2 Influence of the mesh size and time step

To assess the dependency of the solution on the mesh size and time step, we simulated the reference case from the validated solver on three different mesh sizes (finest level size 20.4, 15.1, 12.0 μm), and 5–6 different time steps. The time steps were chosen such that the coarsest step always led to a maximum Courant–Friedrichs–Lewy (CFL) number close to (but below) 1 (i.e. the fluid moves less than a grid step in a single time step). In the tested range, mesh size nor time step had a large influence on the solution.

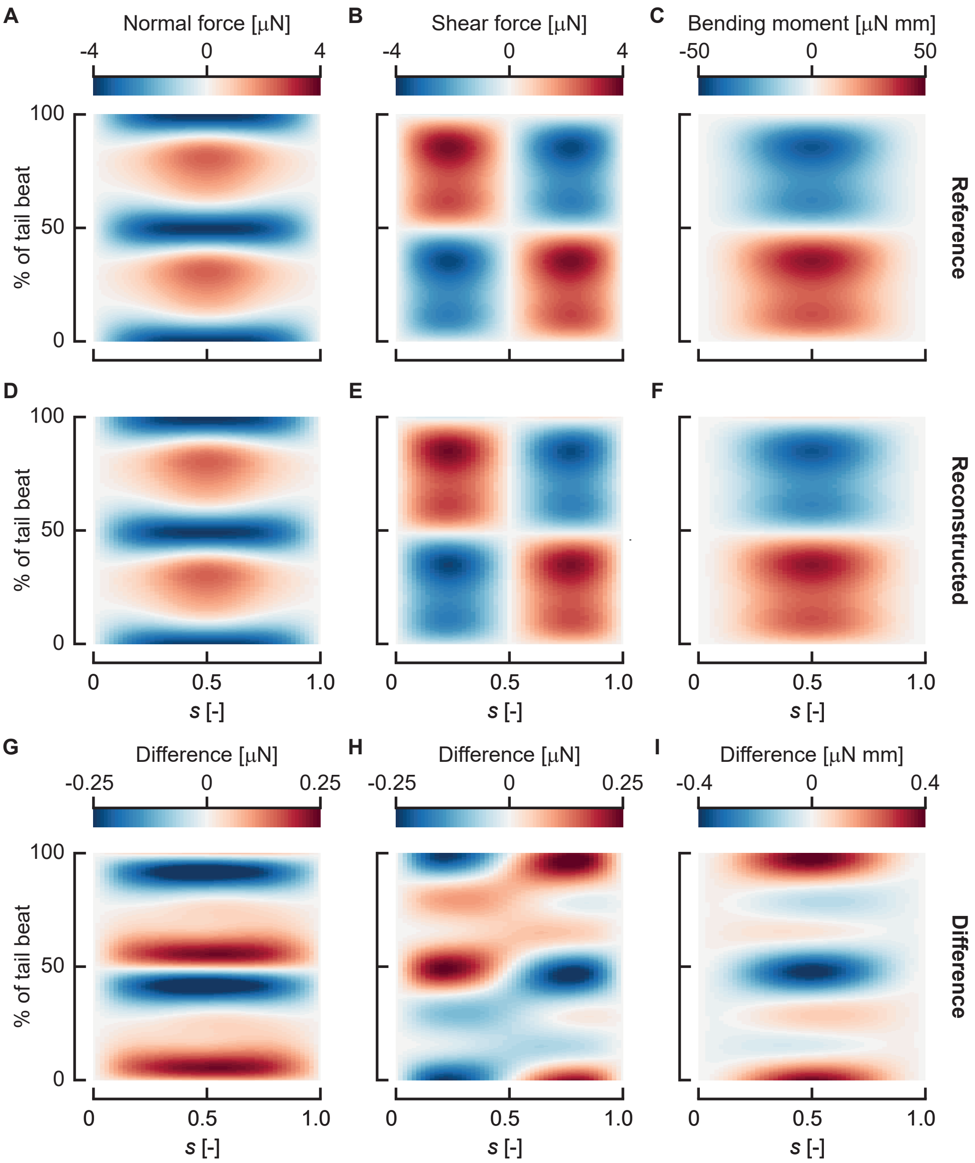

Fig. S3. Comparison between reference and reconstructed internal forces and moments. (A)–(C) Reference normal force (A), shear force (B), and bending moment (C). (D)–(F) Reconstructed normal force (D), shear force (E), and bending moment (F). (G)–(I) Difference between the reference and the reconstructed normal force (G), shear force (H), and bending moment (I).

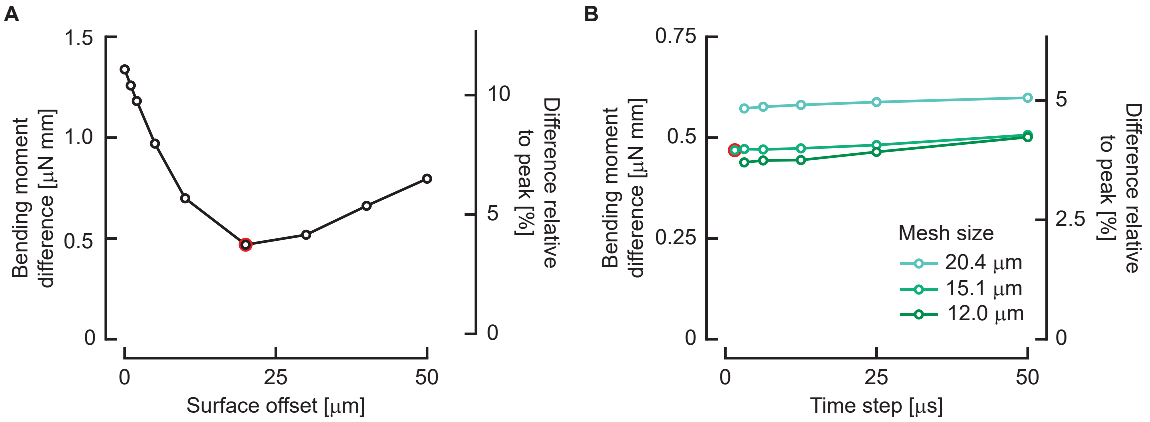

Fig. S4. Convergence of the immersed boundary solver. (A)–(B) The difference in reconstructed bending moment compared to the reference solution as a function of the offset of the surface used for force calculations ((A)), and the mesh size and time step ((B)). The final choices of surface offset, mesh size, and time step size are highlighted with a red circle. The underlying data for this figure can be found in S2 Data.

For mesh size, the largest step in accuracy is from 20.4 μm to 15.1 μm, the step to 12.0 μm is smaller. We chose a mesh size of 15.1 μm, as it allows reasonable accuracy while remaining computationally feasible—memory usage is a limiting factor on our computational facilities as the meshes get larger. For the time step, smaller time steps lead to marginally smaller errors. Computation times are at most linearly affected by the time step, so the trade-off for computational feasiblity is less relevant. Hence, to be on the safe side, we chose a time step of 0.5 μs for solving the fluid dynamics.

A comparison of the reference simulation with the final choice of mesh size 15.1 μm and time step 0.5 μs is shown in Fig. S5. The bending moments show qualitatively similar patterns, in time and space. The magnitude is slightly underestimated in IBAMR compared to the reference solution. Considering the very different approach between the two CFD solvers (overset meshes vs. immersed boundary method), we do not expect the solutions to overlap perfectly—both contain different sources of error. However, the excellent qualitative and good quantitative overlap between the two solutions provides confidence that major modelling or numerical errors are absent.

7 Data availability

Data and source code are made available on Dryad at:

`https://doi.org/10.5061/dryad.2280gb5p6`

Some restrictions apply, because the underlying 'raw' high-speed video data (the unprocessed video frames) exceed the capacity of the repository. These video sequences are stored at a server of Wageningen University. Videos will be made available upon request.

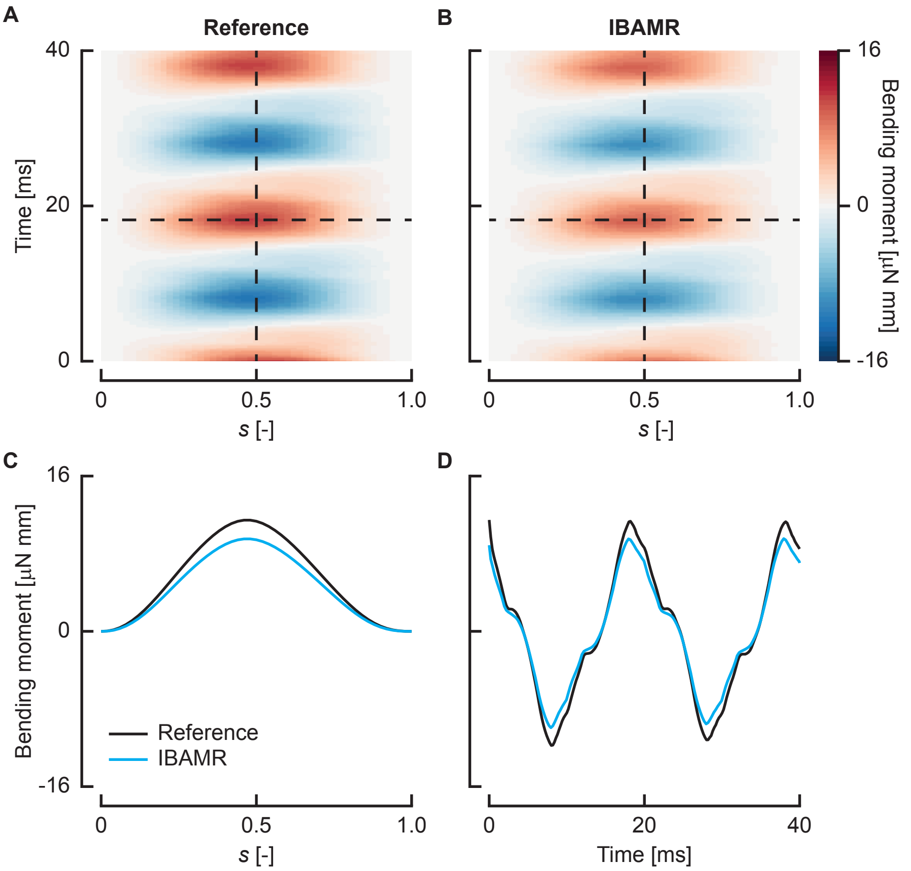

Fig. S5. Comparison between the validated solver and the final choice of immersed boundary solver settings. (A)–(B) The reconstructed bending moment for the fluid dynamic forces from the reference solver (A) and IBAMR (B). The dashed lines indicate the two slices shown in (C) and (D), at a single time step, and a single position along the fish.

References

[1] Voesenek CJ, Pieters RPM, Van Leeuwen JL. Automated reconstruction of three-dimensional fish motion, forces, and torques. PLOS ONE. 2016;11(1):e0146682. doi:10.1371/journal.pone.0146682.

[2] Jones E, Oliphant T, Peterson P, et al.. SciPy: Open source scientific tools for Python; 2001–. Available from: `http://www.scipy.org/`.

[3] Török JS. Analytical mechanics with an introduction to dynamical systems. John Wiley & Sons; 2000.

[4] Oliphant TE. A guide to NumPy. Trelgol Publishing; 2006.

[5] Beléndez T, Neipp C, Beléndez A. Large and small deflections of a cantilever beam. Eur J Phys. 2002;23(3):317. doi:10.1088/0143-0807/23/3/317.

[6] Li G, Müller UK, Van Leeuwen JL, Liu H. Body dynamics and hydrodynamics of swimming fish larvae: a computational study. J Exp Biol. 2012;215(22):4015–4033. doi:10.1242/jeb.071837.

[7] Li G, Muller UK, Van Leeuwen JL, Liu H. Escape trajectories are deflected when fish larvae intercept their own C-start wake. J R Soc Interface. 2014;11(101):20140848–20140848. doi:10.1098/rsif.2014.0848.

[8] Li G, Müller UK, van Leeuwen JL, Liu H. Fish larvae exploit edge vortices along their dorsal and ventral fin folds to propel themselves. J R Soc Interface. 2016;13(116):20160068. doi:10.1098/rsif.2016.0068.

[9] Griffith BE, Hornung RD, McQueen DM, Peskin CS. An adaptive, formally second order accurate version of the immersed boundary method. J Comput Phys. 2007;223(1):10–49. doi:10.1016/j.jcp.2006.08.019.
