# Supplementary material for: Experimental–numerical method for calculating bending moments in swimming fish shows that fish larvae control undulatory swimming with simple actuation
Source: PLoS Biol. 2020 Jul 22;18(7):e3000462. doi: 10.1371/journal.pbio.3000462 (PMC7481021; doi:10.1371/journal.pbio.3000462)
Supplement: Attachment — Submitted filename: vanLeeuwenPLOSBio19.pdf [file pbio.3000462.s003.pdf]

Comments for authors

This manuscript describes an interesting analysis of the forces used by zebrafish larvae during swimming. It takes a mixed experimental and computational approach, with three steps. First, the authors filmed larvae performing fast starts and then swimming. They used three cameras and a 3D matching algorithm that they previously developed to estimate the 3D pose of the larvae, including details of the body shape and deformation. They then used these kinematics and body deformations as inputs to a computational fluid dynamic algorithm to estimate the fluid forces on the body. Finally, they assumed that the body was a homogeneous, linearly elastic beam. They used an optimization algorithm to estimate the internal forces and moments that would be required in such a beam to support the estimated external fluid forces. This three part analysis follows that of Hess and Videler (1984), although the computations are updated with modern algorithms that can address large amplitude 3D movements.

For many tailbeats, they analyze the pattern of bending moments, which is related to the muscle forces, and conclude that the pattern is relatively simple, similar across tailbeats with different speed and acceleration, and similar across development. They also link the bending moments to the speed and acceleration of the tail beat.

The analysis is sophisticated and interesting, although the overall structure of the analysis is not new, and the figures are particularly nice. However, there are four deficiencies in the analysis and the manuscript that will need to be addressed.

1. *Turning*. The most confusing part of this paper is how it does not seem to address turning. The calculated bending moments seem to be left-right symmetric, or at least that is the implication of Fig. 2 and Fig. 4A. However, the authors state that they were filming fast starts (ln. 299), which are a distinctly asymmetric behavior. Did the authors in essence remove any left-right asymmetry by normalizing the bending moment? If so, this should be made much clearer, and justified.
   Even if the normalization is fully explained and justified, I think the authors are leaving out some crucial information by not quantifying turning. The bending moment on one side of the body by itself, or perhaps the difference in left and right side moments, should be much more closely tied to turning parameters than forward swimming speed or acceleration. In contrast, the linear motion parameters that make up the authors' "vigour" metric (velocity and acceleration) should be more closely related to something like the sum or average of left and right side moments.
   I would like to see the authors directly address the symmetry (or asymmetry) of left and right side moments, and how they relate to the linear motion parameters. Without this, I do not think the analysis is complete enough for publication.
   I would also like to see how the left and right side moments relates to turning parameters. This would improve the paper substantially, but would involve substantial additional analysis.

2. *Framing*. I do not see why the authors find it surprising or novel that the actuation patterns are relatively simple. This is how the study is framed in the title, abstract, and introduction, but I do not think it is correct. We have known for a long time that undulatory swimming involves a traveling wave of muscle activity that alternates between left and right sides. This is true in every adult fish studied, with only minor variations, and I see no reason

why larvae should be any different. The physics of fluid motion is nonlinear, but I do not see why that implies that the activation should be anything other than a traveling wave, which is what the authors observe. More generally, the authors suggest that "complex physics" requires sophisticated control, but we know from many different animals that many organisms can move through complicated environments despite having relatively small nervous systems. Even in engineering control, simple feedback controllers can take a nonlinear plant and produce a linear system (see, e.g., chapter 9 in Franklin et al. 2006). I suggest that the authors rethink how they frame their study. I think the most novel aspect of the study is the analysis of acceleration and speed, as shown in Fig. 5.
This paper is also listed as a "Methods and Resource" article, but the authors do not appear to describe any way to get the code to apply these methods to other cases. If the goal of the study is to provide a method, more detail should be given in the main text on the method itself, and the authors should perhaps try applying it to another fish species.

3. *The c parameter*. The authors introduce two new parameters to quantify swimming: "effort", the ratio of bending moment and half-tail-beat duration, and "vigour", which they define as $m(cv^2 + a)$, where m is mass, v is linear swimming velocity, a is linear acceleration, and c is a parameter found by optimizing a linear fit between effort and vigour. In Fig. 5, they compare the components of vigour and the components of effort, and find that they are related. This seems like circular logic.
Is there some way the authors can estimate c in another way? For example, by examining steady tail beats (when $a \approx 0$)?
As the authors state in the discussion, c is related to the drag coefficient. Is there a reason not to define vigour as $\frac{1}{2}\rho S C_D v^2 + m(1 + C_A)a$, where C_D is the authors' previously estimated drag coefficient (ln. 240), and C_A is an added mass coefficient, so that vigour simply represents the sum of the force to overcome drag and the inertial force? This would be more physically justified, in my opinion.
The authors should also address added mass. These are high acceleration behaviors, which almost certainly have high added mass coefficients.

4. *Use of IBAMR*. In general, immersed boundary methods do not simulate predefined kinematics very well. They are designed to simulate fluid-structure interaction problems, in which the internal body forces are specified, but the kinematics evolve as a result of the interaction with the fluid. Several groups have modified IBAMR itself (e.g., Bhalla et al. 2013) or the immersed boundary algorithm (Borazjani et al. 2008) to allow simulations of predefined kinematics, but these modifications are not trivial. How did the authors use IBAMR to simulate fixed kinematics? This needs to be explained in much more detail.

Minor comments

1. I feel that "vigour" is not a very good word for the concept the authors use it for. The dictionary definition is "effort, energy, and enthusiasm", which means it is somewhat synonymous with "effort". I like "effort" as an input parameter, but the authors should think about something other than "vigour" as representing the output. Maybe the "strength" of the behavior?
2. The authors should compare their speed and acceleration results to those of Akanyeti et al. (2017), who analyzed acceleration performance in a wide array of adult fishes, and to Schwalbe et al (2019), who analyzed muscle activity during steady swimming and acceleration in bluegill sunfish.

3. ln. 44. The spinal cord contains quite complicated neural circuits. The fact that it can generate swimming motions does not indicate that the control is simple.
4. ln. 109-110. Kinetic power is the rate of change in kinetic energy of the body (not the fluid), correct? Please clarify.
5. ln. 144. Please justify the normalization more thoroughly.
6. ln. 155. What does "centre of volume of the individual bending moment patterns" mean? Please explain further or write out the equation.
7. ln. 241. The authors argue that because the drag coefficient estimated from the c parameter does not match with another estimate, it means that the equal-cost assumption does not hold, but I think there are a variety of explanations. They could include c being incorrect or not physical, or that part of the cost of acceleration is due to added mass, which is not incorporated in the vigour parameter. Please discuss more thoroughly.
8. ln. 260-264. The authors could approximate the muscle stress in the body, if they assume all of the internal moment comes from muscle. This would provide another way of validating the estimates, by comparing to existing measurements of maximum vertebrate muscle stress.
9. ln. 352-356. With immersed boundary calculations, one can estimate the pressure and velocity gradients on the surface better by incorporating the forces on the IB points themselves. See (Williams et al. 2009).
10. ln. 367. "To determine … equations of motion". I was confused by this sentence until I read the supplemental material. Please try to explain more clearly, defining "control point values".
11. Fig. 4. It would be helpful to provide some frequency distributions for the data. Do fish modulate half-beat duration more often, or peak bending moment?
12. Fig. 5. This figure presents a high dimensional data set, and does it fairly clearly. However, it is hard to distinguish the subtle color differences that encode the output variables (acceleration, speed, vigour). I think it might be better to show acceleration, speed, or vigour as y axis variables, with effort, peak bending moment, or half-beat duration is x axis variables. This would make it possible to see if there are nonlinearities in the input-output relationships, which would address the authors' frame for the paper.
13. Supplemental ln. 94. What is CFL-number? Define
14. Supplemental section 3.1. If I understand the analysis correctly, this is a highly underconstrained optimization problem, which means that multiple optima are possible. How did the authors select a particular optimum?
15. Supplemental Fig. 5C, D. The difference between the reference and IBAMR solutions seems fairly substantial. Please justify further.

Akanyeti O, Putney J, Yanagitsuru YR, Lauder G V., Stewart WJ, Liao JC. 2017. Accelerating fishes increase propulsive efficiency by modulating vortex ring geometry. Proc Natl Acad Sci 114:13828–33.

Bhalla APS, Bale R, Griffith BE, Patankar NA. 2013. A unified mathematical framework and an adaptive numerical method for fluid–structure interaction with rigid, deforming, and elastic bodies. J Comput Phys 250:446–76.

Borazjani I, Ge L, Sotiropoulos F. 2008. Curvilinear Immersed Boundary Method for Simulating Fluid Structure Interaction with Complex 3D Rigid Bodies. J Comput Phys 227:7587–7620.

Franklin GF, Powell JD, Emami-Naeini A. 2006. Feedback control of dynamic systems. 5th ed Upper Saddle River, NJ: Pearson Education.

Hess BYF, Videler JJ. 1984. Fast Continuous Swimming of Saithe (Pollachius Virens): a Dynamic Analysis of Bending Moments and Muscle Power. J Exp Biol 109:229–51.
Schwalbe MAB, Boden AL, Wise TN, Tytell ED. 2019. Red muscle activity in bluegill sunfish Lepomis macrochirus during forward accelerations. Sci Rep 9:8088.
Williams HAR, Fauci LJ, Gaver DP. 2009. Evaluation of interfacial fluid dynamical stresses using the immersed boundary method. Discret Contin Dyn Syst B 11:519–40.
