# Supplementary material for: Experimental–numerical method for calculating bending moments in swimming fish shows that fish larvae control undulatory swimming with simple actuation
Source: PLoS Biol. 2020 Jul 22;18(7):e3000462. doi: 10.1371/journal.pbio.3000462 (PMC7481021; doi:10.1371/journal.pbio.3000462)
Supplement: Attachment — Submitted filename: Authors_reply_to_reviews.pdf [file pbio.3000462.s004.pdf]

Reviewer #1

Comment 1.1

The authors investigate the actuation of fish larvae for undulatory swimming. They develop a model for the dynamics of undulatory swimming and use tracking data from experiments and CFD to estimate the external hydrodynamic forces and deduce the distribution of internal bending moments. Similar inverse dynamics approaches have been used in previous studies, and are cited appropriately. The approach presented here is detailed and robust and supports the analysis of a large dataset. The study yields new interesting insight into the actuation dynamics. I only have a few comments.

Reply 1.1

We thank the reviewer for the positive and accurate summary of our work, and the constructive review. It is correct that previous studies have used similar approaches, but our newly-developed method is the first to implement a combination of automatic tracking and bending moment computations for large amplitudes with a full numerical solution of the Navier Stokes equations in 3D. Below, we address all comments in detail.

Comment 1.2

1. *The authors define "vigour". The definition seems a bit arbitrary and I don't think the vigour physically represents anything, or else it should be explained. The effort on the otherhand, seems to be the more physical quantity, and relates to a maximum power input (also units of effort in fig 5 are given in Newtons, but should be in units of Watts). Why do the authors not use effort or the mean resultant power in their analysis on figure 4.*

Reply 1.2

The reviewer is correct that the metrics *effort* and *vigour* are correlated, but they are conceptually different: *effort* represents the "input" of the fish when performing a tail beat, while *vigour* represents the "output" resulting from the invested effort. The effort is directly (analytically) related to the output quantities in Fig. 4G, H, so the results would merely be an analytical function. On the other hand, the vigour is only indirectly dependent on the "input" (via the bending moment distributions), and hence the more interesting quantity to analyse.

To also explain this difference better in the manuscript, we added a sentence to the results (lines 162–164) and expanded the discussion section on the physical meaning of the vigour (lines 324–335). Here, we now also have added a section about the relative effect of body drag and added mass on vigour.

Thank you for spotting the erroneous unit in Fig. 5. This has been corrected.

Comment 1.3

2. *Related to point 1./. Fig 3 c.d. seem to suggest some sort of bimodal distribution of larvae between larvae that accelerate strongly and swim slowly and larvae that swim faster but don't accelerate so strongly. It seems that the definition of vigour collapses the two artificially, but these modes*

should be different in their actuation. While these two modes may be similar in the spatial distribution of the actuation (fig.4 cd), they may be different in the time distribution of the actuation (fig. 4ef only represents the phase of maximum bending). These differences may be characterized in fig 2 c.d., by looking at the time distribution of curvature and moment.

Reply 1.3

This is an intriguing observation. We therefore tested whether we could separate tail beats into those for high-speed swimming and high-acceleration swimming, or whether the bending moment distributions were similar across speeds and accelerations.

To explore this, we characterised the difference between all bending moment distributions over space and time not only by comparing the spatiotemporal phase (as shown in Fig. 4), but also by calculating the mean absolute difference of each point to the corresponding point in the mean distribution. These differences appeared to be small, indicating that the distributions are similar across different swimming speeds and accelerations (i.e. our "vigour"). We rephrased this in the results to make this more clear (lines 209–212).

Comment 1.4

3. *The supplemental information contains most of the useful information, to understand the approach. It could benefit from minor reorganization, as reading through was at times confusing because of the sequence.*

Reply 1.4

We reframed the article to provide a better fit with the category of a "PLOS Biology Methods and Resources" article. During this reorganisation, we moved several paragraphs from the Supporting Information to the main text and reordered and rephrased them.

Reviewer #2

Comment 2.1

This study is certainly impressive and thorough methodologically. My comments therefore focus on the broader scientific messages, most of which should be simple to deal with in revision.

Reply 2.1

We thank the reviewer for the positive assessment of our developed methodology, and for the detailed and constructive feedback. As explained below, we have incorporated all your suggestions and comments in the revised manuscript.

Comment 2.2

1. *Is the hypothesis as expressed worthwhile? Or is it actually almost circular? The hypothesis (L.14) can be summarized as: a simple brain results in simple actuation. Is this not a (sorry) no-brainer? If it is the brain that initiates activation, is it not part of the definition of a simple brain that it should only be capable of simple activation?*

Reply 2.2

The question that we intend to solve is not necessarily whether the larvae use a simple actuation pattern. Instead, we wondered how these larvae, despite their simple brains, can control their swimming speed and acceleration, and how they can maintain swimming control throughout their rapid development.

We rephrased the abstract and introduction to more accurately reflect the intent of the article—focusing more on the adjusting of swimming speed and changes throughout development, rather than the (simple) production of swimming motion. In addition, we now put more focus on the methodological innovations presented, considering the "methods and resources" status of the article.

Comment 2.3

2. *Is the result notable or surprising? (And does it matter if it is not?) L. 78-84. That amplitude and frequency determines things sounds pretty intuitive. You may wish to expand on why this is not the case, or whether it does not matter that it is intuitive. In effect, your finding could be interpreted as evidence of no change in 'gait'. Similarly, a 'trot' can be used by a horse across a range of speeds and accelerations with only changes in frequency and amplitude. Note that I am NOT saying that your findings are not interesting (after all, why 'should' a fish stick to one 'gait'?). But a small addition or two might prevent the casual reader from being immediately dismissive.*

Reply 2.3

We agree with the reviewer that although some of our results might seem intuitive—which they are not necessarily—it is worthwhile to confirm them with evidence. In addition, we do not only show that swimming action is similar at different speeds and accelerations, but also remains strikingly similar throughout development. This is less intuitive, because with

increasing size, the fluid characteristics change (expressed by *Re*) and throughout development the neuro-muscular system is rapidly being reorganized. Despite these changes, the swimming actuations remain very similar, illustrating the robustness of the locomotor system.

In the revised manuscript, we rephrased the introduction of our question to more clearly demonstrate what we are answering, and why this is interesting. In addition, to address comments by another reviewer and the editor, we changed the focus of the article more towards the methodology without downplaying the interesting biological results.

Comment 2.4

3. *Does this observation actually require the new methodology? Would (or have) similar conclusions been arrived at from a much simpler kinematic study, or much simpler fluid modeling? Yes, the case is made that previous studies were deficient in some aspect (small/large deflection beam theory, intermediate Re fluids etc.)… but the same can (and indeed you do) be said to some extent about the current study. Inasmuch as all studies and models are deficient in some way, can the case be made for why this one in particular is importantly less deficient? What findings were directly due to the improved methods? What false findings were avoided?*

Reply 2.4

Improvements in the methodology were essential in correctly calculating the internal bending moments of these fish larvae. First, these larvae swim three-dimensionally at large pitch and roll angles—reconstructing their motion in 2D would result in large errors in curvature, and hence in the motion of the propulsive surface through the water. Second, these larvae swim in the intermediate Reynolds number regime. While common simplified fluid-dynamic models work well in low-Re or high-Re regimes, in the intermediate regime they break down [1]. To obtain sufficiently accurate fluid-dynamic forces, we need to solve the full Navier-Stokes equations. Finally, zebrafish larvae can show deformation angles of their centreline of >90°. Previously used models describe the deformation as a purely lateral displacement with respect to the straight fish. These large angles cannot be represented by these models, as the centreline folds over itself, and is hence represented twice at the same x-coordinate. In summary: to analyse the internal bending moments of larval swimming, all methodological advances presented in this article were crucial. We rewrote the introduction and discussion sections of the manuscript to explicitly describe the added value of our new approach compared to previous methods.

Comment 2.5

4. *I suggest being more explicit and earlier about the motivation behind using customized derived metrics (effort and vigor). To what extent is this with the purpose of 'collapsing variation' (L.141) – so effectively being a Principal Component with defined units. And, if a parameter is very highly correlated with power (and, given the units, is this surprising?) why not stick to using*

power? To some extent I feel Figure 3 to be a demonstration that the relationships between force, work, power, and between hydrodynamic and whole-body… all sort of relate intuitively.

Reply 2.5

To clarify our intent and explain the purpose of our custom-derived metrics, we extended the explanation in the results (lines 162–164). Furthermore, we extended the discussion on the physical meaning of the vigour (lines 324–335). For more details about why we use *vigour* and *effort* instead of power, see our reply to *Comment 1.2*.

Minor and line comments

Comment 2.6

There appears to be the implication of an adaptive slant... and this does not feel justified. (L.22 allows function during development).

Reply 2.6

We changed the sentence (line 26–28) to avoid any ambiguities or possible misinterpretation of what we intend to say.

Comment 2.7

The suggestion that complex physics would be (initially) thought to require a sophisticate control system (L. 41) probably overstates matters. Most biologists should be familiar with complex physics occurring with very simple (or zero) control.

Reply 2.7

We rephrased this section to be more accurate.

Comment 2.8

To what extent is the lack of curvature towards the tail tip a consequence of the shape reconstruction? I am not sure how this could be dealt with neatly… but I am suspicious that a 90 degree bend in the last 1% might get smoothed out, whereas the same angle bend at 50% would make for an obviously right-angle fish, and would persist. I don't think this affects the story of the paper, but if it is an inevitable consequence of methodology and not a reliable measurement, this should be noted.

Reply 2.8

Indeed, strong curvatures toward the tail get smoothed out, as discussed in our article that describes the three-dimensional tracking method [2]. However, even a 90° bend in the last 1% moving at the tail beat frequency would make little difference to the fluid mechanics compared to a 90° bend over a length region centred at 50%. The region where curvature is reconstructed most accurately is also the region where curvature changes influence the solution the most. We now mention this in the discussion (lines 292–295).

Reviewer #3

Comment 3.1

This manuscript describes an interesting analysis of the forces used by zebrafish larvae during swimming. It takes a mixed experimental and computational approach, with three steps. First, the authors filmed larvae performing fast starts and then swimming. They used three cameras and a 3D matching algorithm that they previously developed to estimate the 3D pose of the larvae, including details of the body shape and deformation. They then used these kinematics and body deformations as inputs to a computational fluid dynamic algorithm to estimate the fluid forces on the body. Finally, they assumed that the body was a homogeneous, linearly elastic beam. They used an optimization algorithm to estimate the internal forces and moments that would be required in such a beam to support the estimated external fluid forces. This three part analysis follows that of Hess and Videler (1984), although the computations are updated with modern algorithms that can address large amplitude 3D movements.

For many tailbeats, they analyze the pattern of bending moments, which is related to the muscle forces, and conclude that the pattern is relatively simple, similar across tailbeats with different speed and acceleration, and similar across development. They also link the bending moments to the speed and acceleration of the tail beat.

The analysis is sophisticated and interesting, although the overall structure of the analysis is not new, and the figures are particularly nice. However, there are four deficiencies in the analysis and the manuscript that will need to be addressed.

Reply 3.1

We thank the reviewer for the thorough review of the paper and the positive assessment of its quality. In the point-by-point reply below, we address the four major comments as well as the minor comments.

Comment 3.2

1. ***Turning.*** *The most confusing part of this paper is how it does not seem to address turning. The calculated bending moments seem to be left-right symmetric, or at least that is the implication of Fig. 2 and Fig. 4A. However, the authors state that they were filming fast starts (ln. 299), which are a distinctly asymmetric behavior. Did the authors in essence remove any left-right asymmetry by normalizing the bending moment? If so, this should be made much clearer, and justified. Even if the normalization is fully explained and justified, I think the authors are leaving out some crucial information by not quantifying turning. The bending moment on one side of the body by itself, or perhaps the difference in left and right side moments, should be much more closely tied to turning parameters than forward swimming speed or acceleration. In contrast, the linear motion parameters that make up the authors' "vigour" metric (velocity and acceleration) should be more closely related to something like the sum or average of left and right side moments. I would like to see the authors directly address the symmetry (or asymmetry) of left and right side moments, and how they relate to the linear motion parameters. Without this, I do not think the*

analysis is complete enough for publication. I would also like to see how the left and right side moments relates to turning parameters. This would improve the paper substantially, but would involve substantial additional analysis.

Reply 3.1

The reviewer points out an important aspect of our analysis. In our study, we focus on the effect of tail beat kinematics on linear speed and accelerations, and ignore turning dynamics. We chose to do this because the left-right asymmetry of turning is rather more subtle than linear speed and acceleration, and preliminary analyses showed that our current dataset was simply too small and variable in behaviour for systematically analysing the turn dynamics.

The reviewer is correct that many of the analysed tail beats were of turning manoeuvres, and therefore we developed a method that takes this into account: we analysed the motions of the fish per half tail-beat, rather than per full tail-beat. By mirroring all left-half tail-beats, we could analyse the complete dataset without the need to assume that the bending moments are left—right symmetric. This method allowed us to analyse the overall swimming motion in terms of speed and net acceleration, while removing the effects of asymmetries. We rephrased the section "Subdividing motion" to more clearly explain this (lines 446–448).

Although turning dynamics is outside the scope of the current manuscript, we aim to address this in a future study. In the revised manuscript, we therefore now mention turning behaviour as a potential future application of this method. We feel that the current description of the method along with the analysis of linear swimming dynamics meets the goals of a Methods and Resources paper.

Comment 3.3

2. ***Framing.*** *I do not see why the authors find it surprising or novel that the actuation patterns are relatively simple. This is how the study is framed in the title, abstract, and introduction, but I do not think it is correct. We have known for a long time that undulatory swimming involves a traveling wave of muscle activity that alternates between left and right sides. This is true in every adult fish studied, with only minor variations, and I see no reason why larvae should be any different. The physics of fluid motion is nonlinear, but I do not see why that implies that the activation should be anything other than a traveling wave, which is what the authors observe. More generally, the authors suggest that "complex physics" requires sophisticated control, but we know from many different animals that many organisms can move through complicated environments despite having relatively small nervous systems. Even in engineering control, simple feedback controllers can take a nonlinear plant and produce a linear system (see, e.g., chapter 9 in Franklin et al. 2006). I suggest that the authors rethink how they frame their study. I think the most novel aspect of the study is the analysis of acceleration and speed, as shown in Fig. 5. This paper is also listed as a "Methods and Resource" article, but the authors do not appear to describe any way to get the code to apply these methods to other cases. If the goal of the*

study is to provide a method, more detail should be given in the main text on the method itself, and the authors should perhaps try applying it to another fish species.

Reply 3.3

We reframed our study to put more focus on the methodological aspect. We therefore expanded the materials and methods section to provide more detail, which was first present only in the Supporting Information. In addition, we rephrased our aims in the introduction and adapted the discussion accordingly. See also our reply to *Comments 2.2 and 2.3* for more details. Applying our developed methodology to another fish species would require a whole set of new experiments which is impossible given time, cost and labour constraints. Nobody has so far captured the required 3D motion data with sufficient accuracy for a different species.

Comment 3.4

3. ***The c parameter.*** *The authors introduce two new parameters to quantify swimming: "effort", the ratio of bending moment and half-tail-beat duration, and "vigour", which they define as* $m(cv^2 + a)$*, where* m *is mass,* v *is linear swimming velocity,* a *is linear acceleration, and* c *is a parameter found by optimizing a linear fit between effort and vigour. In Fig. 5, they compare the components of vigour and the components of effort, and find that they are related. This seems like circular logic.*

   Is there some way the authors can estimate c *in another way? For example, by examining steady tail beats (when* $a \approx 0$*)?*

   As the authors state in the discussion, c *is related to the drag coefficient. Is there a reason not to define vigour as* $\frac{1}{2}\rho C_D v^2 + m(1 + C_A)a$*, where* C_D *is the authors' previously estimated drag coefficient (ln. 240), and* C_A *is an added mass coefficient, so that vigour simply represents the sum of the force to overcome drag and the inertial force? This would be more physically justified, in my opinion.*

   The authors should also address added mass. These are high acceleration behaviors, which almost certainly have high added mass coefficients.

Reply 3.4

We thank the reviewer for the suggestion to add a more physical justification to the parameter *c*, by modelling drag forces and added-mass forces separately. In the revised manuscript, we now use this suggested approach, and it provided interesting new insights into the relative drag forces and added mass related forces produced by the swimming fish (lines 324–335).

It would be possible to determine *c* based on only (close to) cyclic tail beats, but because the definition of *c* remains unchanged, we expect the results to be similar. We therefore suggest keeping our current analysis method and include the suggested analysis on the relative drag forces and added mass forces in the discussion.

Comment 3.5

4. *Use of IBAMR. In general, immersed boundary methods do not simulate predefined kinematics very well. They are designed to simulate fluid-structure interaction problems, in which the internal body forces are specified, but the kinematics evolve as a result of the interaction with the fluid. Several groups have modified IBAMR itself (e.g., Bhalla et al. 2013) or the immersed boundary algorithm (Borazjani et al. 2008) to allow simulations of predefined kinematics, but these modifications are not trivial. How did the authors use IBAMR to simulate fixed kinematics? This needs to be explained in much more detail.*

Reply 3.5

IBAMR provides an interface (in C++) to prescribe kinematics of the Lagrangian points directly, either as positions or velocities of these points. We developed a (non-trivial) add-on to IBAMR that provides the kinematics of our zebrafish larvae through this interface. In the materials and methods section of the revised manuscript, we now describe this add-on in more detail (section "Calculating fluid-force distributions" in the Methods). In addition, the add-on code including instructions for its usage are available in the digital repository linked to the paper (section Data Availability).

Minor comments

Comment 3.6

1. *I feel that "vigour" is not a very good word for the concept the authors use it for. The dictionary definition is "effort, energy, and enthusiasm", which means it is somewhat synonymous with "effort". I like "effort" as an input parameter, but the authors should think about something other than "vigour" as representing the output. Maybe the "strength" of the behavior?*

Reply 3.6

We thank the reviewer for this suggestion; however, we are not certain that "strength" is a less ambiguous term. We feel that "strength" has a stronger connotation with physical concepts (e.g. force, power) than does "vigour". Furthermore, one of the definitions from the Merriam-Webster dictionary ""*intensity of action or effect*"" fits the intent quite well.

Comment 3.7

2. *The authors should compare their speed and acceleration results to those of Akanyeti et al. (2017), who analyzed acceleration performance in a wide array of adult fishes, and to Schwalbe et al (2019), who analyzed muscle activity during steady swimming and acceleration in bluegill sunfish.*

Reply 3.7

We thank the reviewer for this suggestion; we now briefly discuss these articles in the light of the present observations (lines 371–378).

Comment 3.8

3. *ln. 44. The spinal cord contains quite complicated neural circuits. The fact that it can generate swimming motions does not indicate that the control is simple.*

Reply 3.8

We removed this sentence.

Comment 3.9

4. ln. 109-110. Kinetic power is the rate of change in kinetic energy of the body (not the fluid), correct? Please clarify.

Reply 3.9

We rephrased this sentence for clarity (lines 146–147).

Comment 3.10

5. *ln. 144. Please justify the normalization more thoroughly.*

Reply 3.10

In the revised manuscript, we have expanded our reasoning for normalising the bending moments (lines 191–193).

Comment 3.11

6. *ln. 155. What does "centre of volume of the individual bending moment patterns" mean? Please explain further or write out the equation.*

Reply 3.11

We now provide more detail on the calculation (lines 217–221).

Comment 3.12

7. *ln. 241. The authors argue that because the drag coefficient estimated from the* c *parameter does not match with another estimate, it means that the equal-cost assumption does not hold, but I think there are a variety of explanations. They could include* c *being incorrect or not physical, or that part of the cost of acceleration is due to added mass, which is not incorporated in the vigour parameter. Please discuss more thoroughly.*

Reply 3.12

We expanded the discussion on the c-parameter, see also our reply to *Comment 3.4* for more details.

Comment 3.13

8. *ln. 260-264. The authors could approximate the muscle stress in the body, if they assume all of the internal moment comes from muscle. This would provide another way of validating the estimates, by comparing to existing measurements of maximum vertebrate muscle stress.*

Reply 3.13

We thank the reviewer for this suggestion. Determining the muscle stress from only the net bending moment is an indeterminate problem—infinitely many different solutions exist to obtain the same net bending moment. To solve reliably, we need additional measurements (e.g. temporal-spatial muscle activation patterns, which are extremely difficult to obtain), or a muscle model. Both extra measurements and further modelling are outside the scope of this article, and without them we cannot calculate an accurate estimate for the peak muscle stress.

Comment 3.14

9. *ln. 352-356. With immersed boundary calculations, one can estimate the pressure and velocity gradients on the surface better by incorporating the forces on the IB points themselves. See (Williams et al. 2009).*

Reply 3.14

The motion we prescribe is not divergence-free, which led to issues in the velocity field inside the fish. For this reason, we describe only the surface of the fish with Lagrangian points. As a result, there is an "internal" flow inside the fish which disturbs the net force distribution on the surface. For this reason, we calculate the force distribution from the external flow field (which is not influence by the spurious internal flow field). Because the Reynolds number is relatively low, velocity gradients are quite limited, so the small offset from the surface does not substantially influence the result. We expanded the explanation for our approach and why it was necessary (lines 498–506).

Comment 3.15

10. *ln. 367. "To determine … equations of motion". I was confused by this sentence until I read the supplemental material. Please try to explain more clearly, defining "control point values".*

Reply 3.15

We addressed the reviewer's comment by expanded this section considerably (section "Calculating bending moments" in the Methods).

Comment 3.16

11. *Fig. 4. It would be helpful to provide some frequency distributions for the data. Do fish modulate half-beat duration more often, or peak bending moment?*

Reply 3.16

We thank the reviewer for the interesting suggestion; we added two extra panels to Fig 5 showing the frequency distribution of peak bending moment and half-beat duration for low, medium, and high effort. We explained these panels in lines 259–266 and discussed the change in relative contribution of half-beat duration and peak bending moment with increasing effort in lines 386–390.

Comment 3.17

12. *Fig. 5. This figure presents a high dimensional data set, and does it fairly clearly. However, it is hard to distinguish the subtle color differences that encode the output variables (acceleration,*

speed, vigour). I think it might be better to show acceleration, speed, or vigour as y axis variables, with effort, peak bending moment, or half-beat duration is x axis variables. This would make it possible to see if there are nonlinearities in the input-output relationships, which would address the authors' frame for the paper.

Reply 3.17

We thank the reviewer for the feedback on the visualisation of our data set. In making this figure, we tried several permutations of the axes, and concluded that having the "input" variables on the axes and the "output" colour-coded showed the clearest result. In addition, Fig. 3C and D show the data in this fashion, against effort on the x-axis. The new information in Fig. 5 is the subdivision in peak bending moment and half-beat duration, which we concluded to be the main input parameters based on the analysis of Fig. 4. However, we added two additional panels showing the frequency distribution of the peak bending moment and the half-beat duration (see Reply 3.16).

Comment 3.18

13. Supplemental ln. 94. What is CFL-number? Define

Reply 3.18

We moved this section to the main text and added an explanation of the CFL-number (lines 490–491).

Comment 3.19

14. Supplemental section 3.1. If I understand the analysis correctly, this is a highly underconstrained optimization problem, which means that multiple optima are possible. How did the authors select a particular optimum?

Reply 3.19

For several test cases, we tried to initialise optimisation from any particular frame from the previous frame, from a distribution of zeros, and from random distributions. All these different initialisations converged to the same solution—the problem is relatively insensitive to the initial conditions. In addition, our validation of the method reproduces the reference internal forces and moments almost perfectly from the same information as the real dataset: motion and external force. This provides further confidence in the reliability of the method. We now mention this in the revised manuscript (lines 560–561).

Comment 3.20

15. Supplemental Fig. 5C, D. The difference between the reference and IBAMR solutions seems fairly substantial. Please justify further.

Reply 3.20

We expanded the explanation of the differences. (section 6.2 in S1 Text).

References

1. Van Leeuwen JL, Voesenek CJ, Müller UK. How body torque and Strouhal number change with swimming speed and developmental stage in larval zebrafish. J R Soc Interface. 2015;12: 20150479. doi:10.1098/rsif.2015.0479

2. Voesenek CJ, Pieters RPM, Van Leeuwen JL. Automated reconstruction of three-dimensional fish motion, forces, and torques. PLoS One. 2016;11: e0146682. doi:10.1371/journal.pone.0146682

3. Akanyeti O, Thornycroft PJM, Lauder G V, Yanagitsuru YR, Peterson AN, Liao JC. Fish optimize sensing and respiration during undulatory swimming. Nat Commun. 2016;7: 11044. doi:10.1038/ncomms11044

4. Schwalbe MAB, Boden AL, Wise TN, Tytell ED. Red muscle activity in bluegill sunfish Lepomis macrochirus during forward accelerations. Sci Rep. 2019;9: 1–13. doi:10.1038/s41598-019-44409-7
