# Supplementary material for: Experimental–numerical method for calculating bending moments in swimming fish shows that fish larvae control undulatory swimming with simple actuation
Source: PLoS Biol. 2020 Jul 22;18(7):e3000462. doi: 10.1371/journal.pbio.3000462 (PMC7481021; doi:10.1371/journal.pbio.3000462)
Supplement: Attachment — Submitted filename: vanLeeuwenPLOSBio19R1.pdf [file pbio.3000462.s005.pdf]

Comments for authors

This is a nice manuscript, with sophisticated and important analysis. I would like to see it published. However, the authors have incompletely addressed many of my criticisms. I would like to see more changes in the text to address my previous criticisms.

1. *Turning*. At a minimum, in the methods (around ln. 453) and results (around ln. 158), the authors should explain in more detail how they calculate acceleration. At any time interval, even in the horizontal plane, there are at least two relevant acceleration magnitudes – parallel to the current heading and perpendicular. I think it is likely that the different accelerations rely on different mechanisms, and the authors should discuss this more thoroughly.

More broadly, I think the authors are missing an opportunity, even if the focus in this paper is on linear motion, to analyze fundamental mechanisms. The ratio of the magnitude of internal moments in this half tail beat to the previous one (in other words, the ratio of left side moments to right) is probably strongly related to linear swimming performance. When they are more equal, the linear swimming performance is probably higher. The authors could easily plot vigour relative to this ratio.

2. *c parameter*. The authors have discussed the *c* parameter better in the Discussion, but it still needs a better motivation in the Results (lns. 178-184). They also have not addressed my fundamental criticism of the *c* parameter, which is that the logic is circular. In Fig. 3, they determine the *c* parameter by a linear fit of vigour to effort (the ratio of peak bending moment and half-beat duration), then in Fig. 5, they that vigour is strongly related to effort. I think the authors need a way to independently estimate the *c* parameter, if they're going to use it to make the arguments shown in Fig. 5.

3. *Control parameters*. This is a new criticism, and I apologize that I did not recognize it when I first read the paper. There is one more control parameter that the authors have not analyzed, but which might be important: the wavelength of the bending moment distribution. Even with the left-right normalization, there is a distribution of bending moments along the body. It may be appropriate to ignore that, in favor of focusing on peak moment and timing of the peak, but the authors should justify carefully. Previous studies of acceleration performance have found that wavelength changes during acceleration (eg, Schwalbe et al. 2019).

Minor comments

1. Fig. 2F. Are there any times when the fluid power is negative (ie, the animal is extracting energy from the fluid)? If so, it would be nice to highlight those clearly on the figure, and maybe also discuss in the manuscript.
