# Supplementary material for: Experimental–numerical method for calculating bending moments in swimming fish shows that fish larvae control undulatory swimming with simple actuation
Source: PLoS Biol. 2020 Jul 22;18(7):e3000462. doi: 10.1371/journal.pbio.3000462 (PMC7481021; doi:10.1371/journal.pbio.3000462)
Supplement: Attachment — Submitted filename: Response_to_reviews_final.pdf [file pbio.3000462.s006.pdf]

Academic Editor

Comment AE.1

Our Academic Editor wishes to emphasise the need to compare with previous methods and to make further effort to reframe your study as a Methods and Resources paper in your revision. We would also like to stress reviewer 3's concern regarding a possible circularity in your use of your "c parameter". Furthermore, you will need to make your code available. We cannot make any decision about publication until we have seen the revised manuscript and your response to the reviewers' comments. Your revised manuscript is also likely to be sent for further evaluation by the reviewers.

Reply AE.1

We thank the editors for giving us the opportunity to revise our manuscript. In our revised manuscript, we have addressed all comments by the editor and reviewers, as we describe here in our point-by-point response document. The academic editor emphasised four points of attention that we addressed as follows:

1. *The need to compare with previous methods*. In response to a suggestion by Reviewer #2, we have added a comparison between the present method and a previous small-amplitude modelling approach. This comparison explicitly shows the striking increase in accuracy of our new model. See **Reply 2.1** below for more details.

2. *Reframing the study as a Methods and Resources paper*. As requested by the editor, we further reframed our manuscript as a Methods and Resources paper. Most importantly, we extended the Results chapter with (1) a section that summarises our newly developed method in more detail, including a description of the models used and (2) a new "method testing and validity" sub-section that includes model validations and the new comparison with previous methods. Following these sections, we describe the results of our larval fish analysis, which serves as an example application of the method.

3. *Possible circularity in the use of the "c parameter"*. We rephrased potentially misleading sentences in response to Reviewer #3's criticism, hopefully addressing it adequately. See **Reply 3.4** below for more details.

4. *The need to make our code available*. The complete source code of our newly developed method for modelling fish swimming will be made available in a Dryad repository upon acceptance.

Reviewer #1

The authors have addressed the comments satisfactorily.

Reviewer #2

Comment 2.1

This revision reads nicely and appears appropriate as a Methods and Resources Article.

My single major comment is that perhaps a new methodology should be compared with the older ones. Fine, the case is made that it is bound to be better… but how different, and when are the differences most significant? And which of the additions make the difference?

If we could start off with the 'oldest' of small-angle beam theory, simplified 2-d and steady-state, could some quantification be made of the changes due to the 'newest' of large-deflection beams, modern 3-d CFD, unsteady? Even if it was just looking at the extreme cases of biggest deflection etc.… just how different (let us assume wrong) would the old methods give?

Reply 2.1

We agree with the reviewer that a direct comparison of our "large amplitude" fish model with the older "small amplitude" models is very valuable. We initially refrained from this, because it required us to entirely reimplement the "small-amplitude" beam model in a validated software package, as no source code for these models is available.

However, we agree with the reviewer that without such direct comparison, one cannot assess how large the improvement of our new model is, and therefore we now reimplemented the small-amplitude beam model used in previous studies [1–3], and performed the direct comparison with the present large-amplitude beam model. This approach highlights the advantages of the large-amplitude model, disentangling it from additional effects of the fluid-dynamic assumptions. See the Supporting Information section 6, and Figs. S4,S5 for more details.

In this comparison, we applied both beam models to the synthetically generated reference data that we had previously used to validate our bending moment reconstruction method. This allowed us to systematically test how both models perform for fish swimming at various deformation amplitudes. The results show that at small amplitudes, the small-amplitude model already produces larger errors in reconstructed bending moments than the large-amplitude model. These errors rapidly grow with increasing amplitude—even for moderate deformation amplitudes, the reconstructions error in bending moment can already be about 40%. In addition, the small-amplitude model shows considerable phase shifts of peaks in the bending moment. The curvature amplitudes made by fish during fast starts are even larger than those considered in the model comparison; they cannot be analysed reliably with the small-amplitude model. We modified the main text to more thoroughly address these two improvements (lines 65–69).

Next to the new beam model, there are two additional major differences between the present method and previous methods: the ability to model aperiodic swimming, and the fluid dynamic model. In section 6 of the Supporting Information, we discuss the advantages of

dropping the assumption of periodicity. In the Methods section (lines 624–629), we discuss the advantages of using full computational fluid dynamics rather than an inviscid model to predict fluid dynamic forces in the intermediate Reynolds number regime.

Very minor comments

Comment 2.2

I presume you are avoiding discussion of various 'efficiency' terms for a reason… it probably falls outside the scope of this paper; if not, around Line 145 might be a place to comment.

Reply 2.2

The reviewer is correct in his/her assertion that we do not want to make claims on energetics based on the bending moments, as we cannot reliably do so (see also section 3.2 of the Supporting Information).

Comment 2.3

Line 178. Reynolds numbers aren't actually given from what I can see (merely that they fall in an 'intermediate' regime). But we are thinking that Re is high enough for drag (or resultant force) to be broadly proportional to V^2 not V, aren't we?

Reply 2.3

We now give the range of Reynolds numbers that our experimental data falls in (line 288). The reviewer is correct in their assertion that the Reynolds numbers in our data are far from a purely viscous regime.

Comment 2.4

Passage 375-386 felt a little like a revisiting of something already stated…?

Reply 2.4

While we agree that this passage repeats statements made earlier, we would like to summarise and emphasise them in this section.

Reviewer #3

Comment 3.1

This is a nice manuscript, with sophisticated and important analysis. I would like to see it published. However, the authors have incompletely addressed many of my criticisms. I would like to see more changes in the text to address my previous criticisms.

Reply 3.1

We apologise that we did not address the reviewer's comments to his/her satisfaction in the previous review round. We hope that we have properly addressed the remaining points of critique in this revision. See the point-by-point reply below for more details.

Comment 3.2

1. Turning. At a minimum, in the methods (around ln. 453) and results (around ln. 158), the authors should explain in more detail how they calculate acceleration. At any time interval, even in the horizontal plane, there are at least two relevant acceleration magnitudes – parallel to the current heading and perpendicular. I think it is likely that the different accelerations rely on different mechanisms, and the authors should discuss this more thoroughly.

More broadly, I think the authors are missing an opportunity, even if the focus in this paper is on linear motion, to analyze fundamental mechanisms. The ratio of the magnitude of internal moments in this half tail beat to the previous one (in other words, the ratio of left side moments to right) is probably strongly related to linear swimming performance. When they are more equal, the linear swimming performance is probably higher. The authors could easily plot vigour relative to this ratio.

Reply 3.2

We expanded the explanation in the main text on how we calculate acceleration (line 465–467); we use a simple definition that only takes into account the change in speed.

We agree with the reviewer that accelerations that cause turning and change in speed might be achieved using different swimming mechanisms, and that there are plenty more interesting avenues of research with this method. However, given that this is a Methods and Resources article, we would like to limit the scope of the results to the relatively simple, focussed analysis that we have done so far. In future analyses, we and other researchers can apply our method to study swimming mechanisms like the one suggested by the reviewer. To this end, we provide the full data set and source code of all programs developed in this article.

Comment 3.3

2. c parameter. The authors have discussed the c parameter better in the Discussion, but it still needs a better motivation in the Results (lns. 178-184).

Reply 3.3

We expanded the explanation and motivation of our definition of vigour and the usage of the parameter *c* (lines 196–198).

Comment 3.4

They also have not addressed my fundamental criticism of the c parameter, which is that the logic is circular. In Fig. 3, they determine the c parameter by a linear fit of vigour to effort (the ratio of peak bending moment and half-beat duration), then in Fig. 5, they that vigour is strongly related to effort. I think the authors need a way to independently estimate the c parameter, if they're going to use it to make the arguments shown in Fig. 5.

Reply 3.4

We modified the relevant results section to clarify that the increase in *vigour* with *effort* is a result of our definitions (line 249). The remainder of the claims in this section (and the

Discussion below) concern the speed and acceleration individually, and hence are not influenced by the definition of vigour based on effort.

Comment 3.5

3. Control parameters. This is a new criticism, and I apologize that I did not recognize it when I first read the paper. There is one more control parameter that the authors have not analyzed, but which might be important: the wavelength of the bending moment distribution. Even with the left-right normalization, there is a distribution of bending moments along the body. It may be appropriate to ignore that, in favor of focusing on peak moment and timing of the peak, but the authors should justify carefully. Previous studies of acceleration performance have found that wavelength changes during acceleration (eg, Schwalbe et al. 2019).

Reply 3.5

This is an interesting suggestion, and so we looked further into it; this showed that the wavelength of the bending moment is similar between all motions. This is most apparent in in Fig. 4 of the manuscript: the bending moment distributions are similar across development and swimming vigour, and as a result only the peak bending moment and tail beat duration are left as control parameters. In the revised manuscript, we now mention this explicitly (lines 250).

Minor comments

Comment 3.6

1. Fig. 2F. Are there any times when the fluid power is negative (ie, the animal is extracting energy from the fluid)? If so, it would be nice to highlight those clearly on the figure, and maybe also discuss in the manuscript.

Reply 3.6

We thank the reviewer for their interesting suggestion. Further investigation shows that only a very small fraction of the points (approximately 1%) attains small negative values (approximately 1% of the maximum power), while the rest is positive. This falls within the range of error; it appears that fluid power does not attain considerable negative values.

References

1. Hess F, Videler JJ. Fast continuous swimming of saithe (*Pollachius virens*): a dynamic analysis of bending moments and muscle power. J Exp Biol. 1984;109: 229–251.

2. Cheng J-Y, Pedley TJ, Altringham JD. A continuous dynamic beam model for swimming fish. Philos Trans R Soc B Biol Sci. 1998;353: 981–997. doi:10.1098/rstb.1998.0262

3. Cheng J-Y, Blickhan R. Bending moment distribution along swimming fish. J Theor Biol. 1994;168: 337–348. doi:10.1006/jtbi.1994.1114
