# Supplementary material for: Experimental–numerical method for calculating bending moments in swimming fish shows that fish larvae control undulatory swimming with simple actuation
Source: PLoS Biol. 2020 Jul 22;18(7):e3000462. doi: 10.1371/journal.pbio.3000462 (PMC7481021; doi:10.1371/journal.pbio.3000462)
Supplement: Attachment — Submitted filename: Response_to_reviews_final.pdf [file pbio.3000462.s007.pdf]

Response to reviewers

Reviewer #2

Comment 2.1

I am grateful for the considerable work put in to responding to my previous comments.

The current draft looks very nice. Any wider adoption of the new methods may end being dependent on how easily they can be implemented using the code being made available.

Reply 2.1

We thank the reviewer for his positive comment and previous comments that helped to improve this paper. The source code will be made available on Dryad with appropriate documentation.

Reviewer #3

Comment 3.1

The authors have sufficiently addressed my concerns and the manuscript is ready for publication.

Reply 3.1

We thank the Dr Tytell for his final positive comment and for his previous comments that helped to improve this paper.
